# Autologous fat grafting for cosmetic temporal augmentation: a systematic review

**DOI:** 10.3389/fsurg.2024.1410162

**Published:** 2024-09-20

**Authors:** Sahra Nasim, Henna Nasim, Martin Kauke, Ali-Farid Safi

**Affiliations:** 1 Medical Faculty of the University of Bern, Bern, Switzerland; 2 Department of Surgery, Division of Plastic and Reconstructive Surgery, Yale New Haven Hospital, Yale School of Medicine, New Haven, CT, United States; 3 Center for Craniomaxillofacial Surgery, Craniologicum, Bern, Switzerland; 4 Department of Cranio-Maxillofacial Surgery, Inselspital, Bern University Hospital, Berne, Switzerland

**Keywords:** autologous fat grafting, temporal augmentation, facial rejuvenation, volume retention, patient satisfaction, fat transfer techniques, systematic review

## Abstract

**Background:**

Autologous fat grafting for temporal augmentation is increasingly popular in aesthetic surgery. However, its high absorption rate, unpredictable volume retention rate, and potential safety risks are significant drawbacks. Evaluation methods for the fat graft survival rate, especially volume retention in the temporal area, vary widely and tend to be more subjective than objective. Therefore, this systematic review aims to analyze the unpredictable volume retention rate, associated safety concerns, and the various assessment strategies following autologous fat grafting for cosmetic temporal augmentation.

**Methods:**

We conducted a systematic review of manuscripts listed in the MEDLINE/PubMed database on autologous fat grafting for cosmetic temporal augmentation. Articles had to be available in full text and written in English. Studies not presenting human data or not discussing cosmetic indications were excluded. We adhered to the Preferred Reporting Items for Systematic Reviews and Meta-Analyses (PRISMA) guidelines.

**Results:**

Eight articles were included. The average fat volume injected into each temporal region was 10.69 ml (range 6–17.5) on the right and 10.64 ml (range 5.9–17.4) on the left side. All included articles utilized photographic documentation before and after treatment, along with various questionnaires and scales (37.5% Likert Scale, 12.5% Hollowness Severity Rating Scale, 12.5% Visual Analogue Scale, 12.5% Allergan Temple Hollowing Scale). For objective assessment, one article (12.5%) used computed tomography, and another (12.5%) employed a three-dimensional scanning system to objectively evaluate fat graft survival.

**Conclusion:**

Autologous fat grafting effectively addresses temporal hollowness, with high patient satisfaction and a favorable safety profile. However, the variability in fat retention rates highlights the need for more controlled studies to establish reliable, validated methods for evaluating fat graft survival in the temporal area, and to further assess the safety of this procedure.

## Introduction

Temporal hollowness is caused by a loss of soft tissue volume and elasticity during physiological facial aging or due to pathophysiological alterations after trauma, infection, or tumor surgery (1). Temporal volume loss leads to more prominent bony margins with a concave shape of the upper face region (1). From an aesthetic point of view, this is commonly associated with an unpleasing old and gaunt appearance (1). On the contrary, a youthful face is characterized by a smooth contour of temples (1). Bearing in mind that more and more patients wish to have anti-aging or age-reversing procedures to look more youthful, the restoration of temporal volume is a frequently requested surgical method in daily clinical routine in order to optimize facial aesthetics (2). Injectable fillers, allogeneic implants, autologous grafts, and microvascular transplantation offer the most common and frequently used surgical methods to obtain more temporal volume (3–5). For cosmetic purposes, autologous fat grafting (AFG) is a very well-established technique not only because it is easy to harvest but also because of its abundance, malleability, and low costs it is a commonly used approach for filling and replacement purposes (6). Additionally, the complication rate is low, and the procedure is safe and easy to perform (1). Despite the advantages of AFG and its important role in facial rejuvenation, it is also accompanied by drawbacks. Current studies show a loss of transplanted fat in the temporal area of 20%–90% only 1 year after treatment, resulting in poor aesthetic outcomes and a low patient satisfaction rate (7, 8). This explains why the temporal area seems to be one of the facial subunits with the lowest patient satisfaction rate after autologous fat graft augmentation (8). However, autologous fat grafting is not standardized, and the number of various techniques is relatively high, so existing scientific literature controversially debates, for example, the long-term success rates, especially depending on the applied technique (9). Another major problem is that the evaluation of the clinical outcome, both aesthetically and functionally, is commonly made on subjective assessments by the patient or the investigator, although standardized objective criteria would be advisable to assess this highly delicate procedure. Validated objective assessment methods, such as three-dimensional scanning and imaging systems, radiological evaluation based on computed tomography or magnetic resonance imaging, and standardized questionnaires, would allow filling this gap (1, 10). In order to assess the clinical importance of fat grafting for the restoration of temporal hollowness with objective evaluation tools, we therefore aimed to perform a comprehensive systematic review since, to the best of our knowledge, there is no such existing article. Here, our specific interest lies in elucidating the benefits and drawbacks of the most commonly performed fat grafting techniques, their short- and long-term outcomes, and highlighting future potential developments in this highly demanding facial plastic reconstructive procedure technique.

## Methods

A systematic review of articles on autologous fat grafting for cosmetic temporal augmentation was conducted in December 2021 as previously described (11). We adhered to the Preferred Reporting Items for Systematic Reviews and Meta-Analysis (PRISMA) protocol and performed a MEDLINE database search via PubMed using the following search terms: [(“temporal” or “temple” or “facial”) and (“hollowing” or “depression” or “augmentation”) and (“fat graft” or “grafting” or “fat”)]. We applied the “humans” filter to all searches. Additionally, our search strategy was limited to English articles available in full text.

All relevant articles where study participants underwent autologous fat grafting for temporal augmentation were reviewed. The inclusion criteria were articles presenting original research involving patient data. We included prospective and retrospective studies, case series, and case reports. We excluded review articles, animal or cadaver studies, and articles focused on temporal augmentation for non-aesthetic purposes, such as reconstructive purposes due to, for example, congenital or traumatic temporal volume loss. Furthermore, we excluded studies utilizing other methods for temporal augmentation, such as fillers, flaps, or implants.

**Figure 1:**
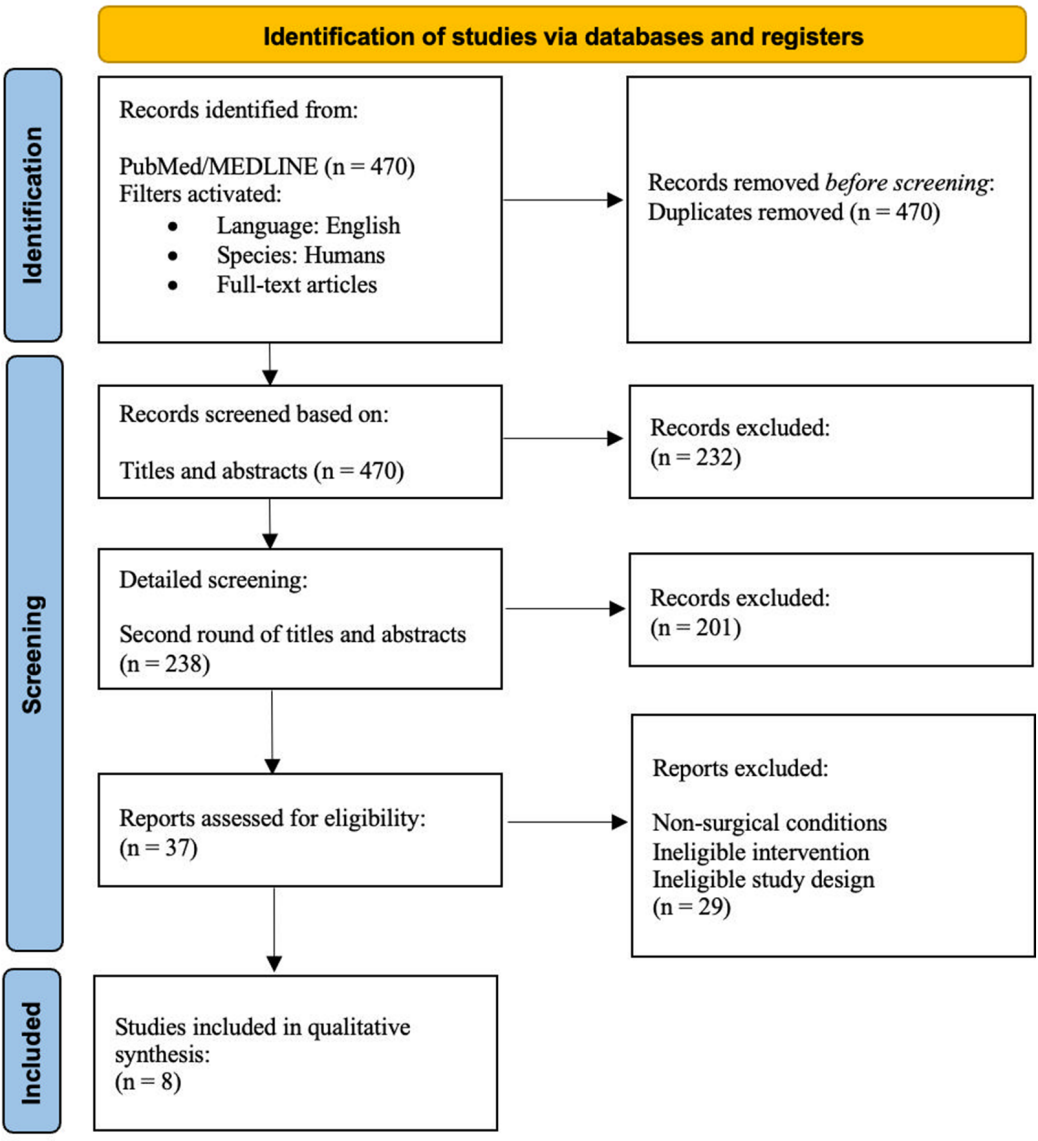
Preferred reporting items for systematic reviews and meta-analyses (PRISMA) flowchart of the selection process to include articles for this systematic review and meta-analysis.

A full-text analysis of the remaining articles was conducted. The PRISMA flowchart (Figure 1) summarizes the exclusion process. We recorded the following data points: authors, year of publication, study design, sample size, amount of volume injected, percentage of volume retention, time to follow-up, subjective and objective assessment criteria, patient satisfaction, and complications.

## Results

For the final manuscript, articles with a total of 719 patients met the inclusion criteria and were included in this systematic review. Table 1 includes a detailed summary of 8 studies on fat grafting for temple augmentation, mostly comprising retrospective reviews with one controlled prospective trial. The studies range in sample size from 34 to 208 patients. Injected volumes per temple range from 6 ml to 657 ml, though not all studies report specific volumes. Volume retention rates are inconsistently reported, but when available, range from 33% to 65%. Follow-up periods vary from 6 months to 96 months.

**Table 1:** Overview of included articles.

Study (year)	Design	Sample size	Fat grafting technique	Donor site	Injected volume per temple (ml)	Volume retention per temple (%)	Follow up period (months)	First follow up after treatment (months)	Objective assessment	Subjective assessment
Lee et al. (12)	Retrospective review	208	Microautologous fat graft transplantation (MAFT-Gun)	Not reported	6.8 (right) 6,5 (left)	Not reported	18	1, 3, 12	Photos	Likert scale
Huang et al. (13)	Retrospective review	96	Targeted fat graft technique	Thigh, abdomen	11.7	65.7	12	12	3D-laser scanning system photos	HSRS Grade by 3 independent observers Satisfaction rate (fully satisfactory, satisfactory, and unsatisfactory)
Chiu et al. (14)	Retrospective review	175	PRP-assisted cell therapy	Abdomen, thigh, knee	NA	NA	12–74	3	Photos	Likert scale
Lin et al. (15)	Retrospective review	39	Microautologous fat graft transplantation	Thigh, abdomen, waist, lateral chest	6.29 (right) 6.34 (left)	NA	20	NA	Photos	HSRS VAS
Diao et al. (16)	Retrospective review	46	Targeted fat graft technique	Thigh, abdomen	10.4	NA	6–24	NA	Photos	Likert scale Allergan Temple Hollowing Scale
Xie et al. (17)	Retrospective review	83	Targeted fat graft technique	Gluteal, abdomen	NA	NA	96	NA	Photos	Satisfaction rate (satisfactory, mostly satisfactory, unsatisfactory)
Hu et al. (2)	Retrospective review	34	Lipoinjection	Upper arm, lower abdomen, thigh	6 (right) 5,9 (left)	33 (right) 32 (left)	12	12	Photos Molded plasticine	Patient's self-evaluation (excellent, good, fair, poor)
Li et al. (18)	Controlled prospective trial	38	SVF-assisted cell therapy	Abdomen, thigh	17,5 (SVF) 16,2 (Fat only)	64,8 (SVF) 46,4 (Fat only)	6	0.25 (POD 7)	Photos CT	NA

SVF, stromal vascular fraction; PRP, platelet rich plasma; 3D, 3-dimensional; CT, computed tomography; HSRS, hollowness severity rating scale; VAS, visual analogue scale; POD, postoperative day.

Outcome measures include both objective and subjective assessments. Objective methods feature photographic analysis, 3D laser scanning systems, and computed tomography (CT). Subjective evaluations include satisfaction rates (ranging from fully satisfactory to unsatisfactory), Likert scales, and self-evaluation by patients. The types of articles included seven retrospective reviews and one controlled prospective trial (2, 12–18).

### Fat grafting techniques

Fat grafting is a well-established method for facial contouring, including cosmetic temporal augmentation. Various approaches have been documented in the literature, focusing on precise fat placement to enhance outcomes. Traditional lipoinjection, a widely accepted technique, involves harvesting fat from donor sites like the abdomen or thighs, processing it, and injecting it into the desired areas. This method allows for effective correction of volume loss in the temporal region and has been extensively used due to its reliability and adaptability.

For instance, Hu et al. utilized traditional lipoinjection for temporal augmentation, where fat was harvested from donor sites, decanted, and injected into the temporal area to correct volume loss (2). Similarly, Chiu et al. described a technique of autologous fat grafting to the temple and forehead, where fat is harvested, processed through centrifugation, and then carefully injected into the target areas (14). Both studies emphasize the effectiveness of traditional fat grafting methods in restoring facial fullness, particularly in the temporal region, highlighting their safety and efficacy in clinical practice.

Some studies have explored more specialized fat grafting methods. For instance, microautologous Fat Transfer-Gun (MAFT-Gun) Using microautologous fat transfer (MAFT). In two articles, the use of MAFT for cosmetic temporal augmentation is described (12, 15). Additionally, Lee et al. used a special device to apply the microautologous fat graft in the temporal fossa, called “MAFT Gun.” The MAFT-Gun can inject specific aliquots of fat precisely into each layer (12). The “3M3l” Technique Huang et al. described the targeted fat graft technique by following the principle of “3M3l”, i.e., Multitunnel, Multilayer, Multipoint, Low-pressure suction, Low Speed, and Low volume (13).

### Adjunctive therapies

Further variations of fat grafting included the use of Stromal Vascular Fraction (SVF)- or Platelet-Rich Plasma (PRP)-assisted cell therapy. Stromal Vascular Fraction (SVF) and Platelet Rich Plasma (PRP) Assisted Cell Therapy Further variations of pure fat grafting are SVF- or PRP-assisted cell therapy for temporal augmentation (14, 18). In the included articles of this systematic review, Li et al. used SVF-assisted fat transfer for cosmetic temporal augmentation (18). After the isolation of SVF from the adipocytes, it is injected into the temporal area combined with the fat graft (18). Chiu et al. combined autologous fat grafting with PRP to restore temporal volume loss (14).

### Temporal augmentation volume and retention rates

The average amount of injected pure fat volume per temple was 9.56 ml (range 6–16.2) on the right side and 9.5 ml (range 5.9–16.2) on the left side (Table 1). The amount of injected volume combined with SVF was 17.5 ml per temple. In only three studies, the evaluation of fat graft retention was part of the follow-up. Huang et al. detected a volume retention rate of 65.7% (13). Hu et al. noted a fat graft survival rate of 33% on the right and 32% on the left side (2). Li et al. described a higher fat graft survival rate by using SVF-assisted fat transfer (64.8% per temple) compared to using a pure fat graft only (46.4% per temple) (18). Most surgeons in the reviewed articles decided the amount of injected volume by their experience and according to the patient's wish (2, 15). Some surgeons subsequently added 30% more fat graft since fat-atrophy after the procedure was already assumed (2, 15, 18).

#### Assessment of volume retention

As part of the follow-up, the assessment of fat graft survival in the temporal area was examined by subjective and objective criteria. The average period between the treatment and the first follow-up was 14 months (range 0.25–26). For subjective evaluation of volume retention, various scales have been used. Patient-rated satisfaction was evaluated by using a Likert scale [very unsatisfied (1), unsatisfied (2), neutral (3), satisfied (4), very satisfied (5)] (12). Chiu et al. used the Likert scale to examine the improvement of temporal hollowness after fat grafting [worse (1)—very much improved (5)] (14). The Likert scale was completed by the patients themselves and by an investigator who was not involved in the operation and was blinded regarding the photographs before and after treatment (14). Huang et al. used the Hollowness severity rating scale (HSRS) consisting of the following grades: 0 = no visible hollowness, 1 = mild hollowness, 2 = moderate hollowness, 3 = severe hollowness (13). The HSRS was completed by three independent observers based on before and after photographs (13). Lin et al. used the HSRS survey to ask the patient to quantify the hollowness severity of the temple before and after the fat grafting treatment (15). Additionally, the Visual analog scale (VAS) was used to evaluate patient satisfaction before and after the treatment. The VAS survey ranges from 0 to 10, where the extreme values indicate no satisfaction and extreme satisfaction, respectively, and 1–3 represent poor, 4–6 fair, and 7–9 excellent satisfaction (15). Diao et al. asked two independent, blinded plastic surgeons with more than 20 years of experience to evaluate the temple hollowing severity based on the photographs before and after treatment according to the Allergan Temple Hollowing Scale, which assigns the grades as convex (0), flat (1), minimal (2), moderate (3), and severe (4) (16). Furthermore, self-created three- or four-point scales have been used to evaluate the satisfaction and/or improvement rate (2, 13, 17). The satisfaction rate (fully satisfactory, satisfactory, unsatisfactory) was classified due to before and after photos by a plastic surgeon who did not operate, by the patient himself, and by an investigator with no medical background (13, 17).

All included studies of this systematic review used photo documentation in a standardized manner for objective assessment of temporal hollowness before and after fat grafting. Hu et al. used molded plasticine preoperatively to evaluate the volume defect in the temporal area (2). Based on the molded plasticine, the amount of fat volume that needed to be aspirated could be estimated (2). By comparing the amount of the molded plasticine to correct the temporal defect, the success of the treatment could be objectively measured (2). Quantitative analysis by using validated imaging/scanning tools like Computed Tomography (CT) and a 3-dimensional (3D) scanning system to evaluate fat graft survival rate in the temporal area have been performed in two studies (13, 18). Huang et al. measured the volume restoration with a three-dimensional (3D) tomographic scan (13). Images of each patient were collected before and (13) months after the fat-grafting procedure (13). Preoperative and postoperative images were overlapped to compare the profile changes according to selected reference points that would not be altered because of treatment (13). The discrepancy between these two images was calculated objectively, and a colored hypsographic image was obtained (13). The survival rate of the fat tissue was defined as Augmentation Volume/(Grafted Fat Volume ×100), and the augmentation rate was defined as Augmentation Volume/(Required Fat Volume ×100) (13). Overall, a total survival rate of 65.7% could be detected 12 months postoperatively (13). Li et al. measured the retention rate in the temporal area by computed tomography (CT) (18). Each patient underwent CT before and 6 months after surgery (18).

#### Complications

Only minor complications were described (Table 2), which were resolved spontaneously or by conservative therapy.

**Table 2:** Complications described in the included articles.

Study	Lee et al. (*n* = 208)	Huang et al. (*n* = 96)	Chiu et al. (*n* = 175)	Lin et al. (*n* = 39l	Diao et al. (*n* = 46)	Xie et al. (*n* = 83)	Hu et al. (*n* = 34)	Li et al. (*n* = 38)
Intraoperative bleeding	No complication was noted during follow up	Not reported	Not reported	10%	None reported	No data reported, but complications were observed in rare cases	Not reported	No complication was noted during follow up
Swelling/seroma		3.1%	98%	Not reported			32.4%	
Headache		7.3%	Not reported	Not reported			Not reported	
Rash/skin irritation		5.2%	Not reported	Not reported			Not reported	
Bruising		Not reported	35%	Not reported			17.6%	
Infection		0%	Not reported	Not reported			0%	
Nodules		0	Not reported	Not reported			5.9%	
Hyperpigmentation donor sites		Not reported	36%	Not reported			Not reported	
Overcorrection		2%	Not reported	Not reported			5.9%	
Undercorrection		Not reported	12%	Not reported			8.8%	
Unevenness		Not reported	3%	Not reported			3%	
Recurrence		Not reported					3%	
Revision/further sessions		2	13					

#### Patient satisfaction

Overall, the reviewed studies show that most patients were satisfied (mean 36%) or very satisfied (mean 52%) with their results (Table 3). One study did not mention patient satisfaction at all.17 Lin et al. evaluated patient satisfaction using VAS and HSRS surveys. The average VAS satisfaction score increased from 4.44 ± 1.33 before the treatment to 8.08 ± 0.77 after the treatment, and the HSRS score dropped from 1.82 ± 0.72 before the treatment to 0.36 ± 0.49 after the treatment.

**Table 3:** Patient satisfaction in the included articles.

Study	Very unsatisfied (%)	Unsatisfied (%)	Neutral (%)	Satisfied (%)	Very satisfied (%)
Lee et al. (*n* = 208)	0	2.4	16.3	42.8	38.5
Huang et al. (*n* = 96)	Not reported	8.3	Not reported	25	66.7
Chiu et al. (*n* = 175)	0	9	17	60	14
Diao et al. (*n* = 46)	0	0	4.3	30.4	65.2
Xie et al. (*n* = 83)	Not reported	2.4	Not reported	14.46	83.13
Hu et al. (*n* = 34)	Not reported	0	5.9	44.1	50
Li et al. (*n* = 38)	Not reported				

## Discussion

Autologous fat grafting (AFG) for cosmetic temporal augmentation is an evolving field in facial rejuvenation surgery since temporal hollowing is a sign of facial aging and ultimately might lead to an aesthetically unappealing appearance (19). Therefore, it is of utmost importance to understand the fundamental mechanical processes of facial aging which lead to temporal volume loss in order to allow adequate and precise treatment of this condition. Rohrich et al. highlighted the eminent role of adipose tissue and in particular its compartments for functional and aesthetic purposes of the face (20). They noted that in a young face, the subcutaneous fat is homogenously distributed, which allows a smooth, full, and convex contour in the temporal area whereas, with physiological aging, the soft and hard tissue volume loss goes along with a disharmonic redistribution of the subcutaneous fat resulting in temporal hollowness (20). However, it is noteworthy to mention that temporal volume loss can also be a result of local and systemic pathophysiological alterations, such as trauma, infection, skeletal growth impairment, oncological surgery, or massive weight loss (1).

In order to gain volume restoration in the temporal area, various techniques exist, such as autologous grafts (bone graft, fat graft) and synthetic filler/implants like hyaluronic acid or alloplastic implants (9, 21, 22). Even though by the use of synthetic material functionally and aesthetically acceptable results can be reached, there are major drawbacks, such as the greater risk of infection, allergenic potential, its necessity of continuous repeating procedures, and its relatively high costs both for practitioners as well as patients (12). Temporal augmentation by using autologous bone grafts is commonly used for the treatment of temporal hollowing after craniotomy (23). Since it is not only an invasive but also a cost- and time-intensive procedure its use seems to be more appropriate for complex aesthetic deformities than for aesthetic temporal augmentation. Autologous fat graft, on the other hand, offers many advantages like abundant availability, perfect biocompatibility, and good cost-efficiency (12).

### Volume retention rates

The main disadvantage is the difficult prediction of resorption and survival rates. Fat graft retention was inconsistently reported and assessed. Huang et al. reported a retention rate of 65.7%, while Hu et al. observed a survival rate of 33% on the right and 32% on the left side (13). Li et al. found that SVF-assisted fat transfer led to a higher survival rate (64.8%) compared to pure fat grafting alone (46.4%) (18). One plausible reason for the low survival rate of fat grafts is the lack of vascularization which results in necrosis and ultimately in a loss of the graft (12). Therefore, different methods have been established to improve fat graft retention. Generally, clinical data on volume retention after temporal augmentation by AFG is limited. A large number of studies depicted that the temporal area is one of the facial subunits with the highest fat resorption rate and the lowest patient satisfaction rate (8). An explanation for the low-fat graft survival rate might be the continuous movement of the temporalis muscle and the relatively avascular nature of the upper and lower temporal compartments (12). Unfortunately, the methods of measuring fat survival rate in the temporal area are seldomly standardized. Indeed, all included articles used pre-and postoperative photographic documentation for objective assessment but only two articles used further objective assessment like a three-dimensional (3D) laser scanning system or computed tomography (CT) to evaluate fat graft survival rate in the temporal area (1, 18). One article used molded plasticine for objective assessment (2). We acknowledge the great importance of photos as an essential and useful tool for a pre-and postoperative documentary but especially for cosmetic purposes, photographs can be misleading due to for example variability of film color, patient's/photographer's position, flash intensity, and facial expression. Therefore, it is of great importance to define objective assessment criteria, which allow reproducible and comparable results. The fact that different and not standardized measurement scales have been used makes the result of the high patient satisfaction not representative. Most articles in our study evaluated fat grafting success in the temporal area just by patient's and/or surgeon's and/or layperson's subjective assessment using various scales like the Likert scale, Hollowness Severity Rating Scale (HSRS), or Visual Analogue scale (VAS) and hereby try to equate postoperative satisfaction results with fat survival rate (1, 12, 14, 16). The effect of fat grafting has been evaluated by nonvalidated questionnaires for many years and objective assessments have relied on pre-and postoperative photographs (14, 16, 17). Even though a large number of objective assessment methods exist, which measure facial fat atrophy, none of them have been used to evaluate systematically fat volume loss in the temporal region. With regards to cosmetic temporal augmentation by AFG, only Li et al. used CT for evaluating fat survival rate in the temporal area (18). Another option for measuring the success of temporal augmentation by using AFG is a three-dimensional scanning system like used by Huang et al. (12). Three-dimensional surface imaging systems are suitable for quantifying fat graft survival rates (24, 25). Magnet resonance imaging (MRI) is also a possible method to measure facial volumetric changes (10). Del Vecchio et al. used MRI to measure volume retention after AFG of the breast and by that they could standardize injection techniques (26).

After systematically reviewing the included articles, we found out that there is also a great lack of investigating the long-term survival rates of fat grafts to correct temporal hollowness. Only one of our reviewed articles considered volume stability after temporal augmentation at a long-term follow-up of 8 years (17). The others (*n* = 7/8) evaluated volume retention only for a short period (6–12 months) and in one study, they followed the patients up for around 24 months.

Very promising options to improve survival rates of autologous fat grafts are from the fields of cell-assisted lipotransfer (27, 28). Our results show that two studies addressed this problem by adding platelet-rich plasma (PRP) or stromal vascular fraction (SVF, Stromal Vascular Fraction) to the transplanted fat graft (14, 18).

While PRP has been investigated as mentioned above, Platelet-Rich Fibrin (PRF) is considered a next-generation platelet concentrate. PRF differs from PRP by its fibrin matrix, which leads to a slower release of growth factors and promotes better tissue repair and angiogenesis. PRF holds potential to further enhance graft retention rates and could offer a more sustained improvement in fat graft survival, though its application in temporal augmentation has yet to be fully explored in the literature. Future studies comparing the efficacy of PRP vs. PRF in cosmetic procedures, particularly in temporal fat grafting, are warranted.

The Stromal Vascular Fraction presents a component of fat tissues which typically includes different cell types such as ASCs, endothelial cells, and other supporting cells with the idea to enhance and improve fat retention and survival (29). Adipose stem cells and activated platelets release several biochemical factors, for example, VEGF (vascular endothelial growth factor) that improve angiogenesis (30). A key limitation of this study is that while SVF-augmented fat grafting shows promise in enhancing fat graft survival, it is not currently approved for clinical use under FDA regulations due to its classification as a drug. This classification requires extensive and costly approval processes, significantly restricting its practical application in routine clinical settings.

In a controlled prospective trial Li et al. demonstrated the benefits of SVF to autologous fat grafts for restoration purposes of temporal hollowness (18). They examined two study groups; 6 out of 12 patients received temporal augmentation with fat graft only and 13 out of 26 individuals received an SVF-assisted fat graft transfer (18). The fat graft survival rate was postoperatively measured by computed tomography (CT) and/or by photographic documentation (18). Overall, fat graft survival was higher with SVF (64.8 ± 10.2%) than fat grafting alone (46.6% ± 9.3%) (18). Yao et al. also analyzed the use of SVF-assisted lipo-injection and the use of fat graft injection only for facial augmentation (27). Compared to the patient group that received lipo-injection only, the patient satisfaction in the SVF-group was significantly higher and the rate of a second surgery to achieve satisfactory results was significantly lower in the SVF-group (27). In the SVF-group 77.3% were satisfied (54.5%) or very satisfied (22.8%) with their results (27). On the contrary, in the patients’ group who underwent lipoinjection only 53.8% were satisfied (48.7%) or very satisfied (5.1%) (27). In contrast to that, the combined use of AFG and PRP seems to make no significant difference in long-term volume retention (31). Fontdevila et al. performed a randomized controlled trial, in which the use of AFG only and AFG with PRP in the therapy of facial human immunodeficiency virus (HIV) atrophy has been investigated (31). The fat volume increase was measured by CT (31). A difference in facial volume gain between both groups could not be confirmed, so the use of PRP was not associated with a better clinical outcome (31). Chiu et al. examined in a retrospective study *n* = 175 patients who underwent temporal and forehead augmentation by using AFG combined with PRP (14). They describe a high patient satisfaction rate of 90% (with 14% very much improved, 60% much improved, and 16% improved) (14). Unfortunately, it is not a controlled study, and the graft survival rate was not objectively measured (14). Therefore it is important, that cell-assisted lipo-transfer for temporal augmentation should gain further attention in the future and its clinical efficacy should be examined by objective measurement methods since current data mostly use subjective assessment criteria (e.g., Likert scale), which tend to have very high risks of patients’ and clinicians’ bias.

### Anatomical considerations and complications

The anatomical complexity of the temporal region poses unique challenges for autologous fat grafting, which can result in suboptimal outcomes such as fat necrosis, irregularities, low retention rates or lead to complications. Huang et al.'s study on temporal fat compartments provides a detailed understanding of these structures, revealing four distinct compartments (Lateral Temporal-Cheek Fat Compartment, Lateral Orbital Fat Compartment, Upper temporal Compartment, Lower temporal compartment) (1). Their findings highlight the importance of precise fat placement using multi-plane injection techniques to ensure safe and effective outcomes in temporal augmentation. One the relevant findings ist the zone of caution (anterior half of the LTC) which includes the temporal branch of the facial nerve, the sentinel vein and middle temporal vessel perforators that can be injured (32).

The studies included in this review reported only a few minor complications like bruising and swelling which revolved spontaneously but overall, no major complications have been reported by using AFG for cosmetic temporal augmentation. However, prior studies in the field have reported on major complications such as blindness, cerebral fat embolism, or pulmonary embolism after accidental intravascular (venous/arterial) fat injection (3, 5, 33, 34). While the procedure may have a low rate of minor complications, it is not without significant risks that must be carefully considered and communicated to patients. In this context, the zone of caution, an area believed to be associated with the highest complication rate, is the anterior half of the lower temporal compartment that contains these crucial neurovascular structures (1). Injection in this region heightens the risk of severe complications. Technically, several precautions are vital to reduce these risks. Avoiding deep injections near the superficial temporal artery and using blunt cannulas, which reduce the likelihood of vascular injury, are essential steps. Slow, low-pressure injections with constant cannula motion help prevent fat from entering the bloodstream as high pressure can lead to retrograde arterial injection. One can aspirate to assure that the cannula is not intravascular. Additionally, understanding and recognizing the early signs of complications, such as visual disturbances, enables rapid intervention, potentially averting serious outcomes like blindness.

Other complications can be related to the technique of fat injection. For example, high pressure injection and over injection of fat volume in one location can limit diffusion of oxygen and nutrition in the early phase and impair neovascularization (35). This is because grafts rely on the recipient site blood supply and plasmatic imbibition in the early phases. Consequently, cells can necrose with reduced volume retention and possibly oil cyst formation which may lead to contour irregularities. In addition to optimizing harvest, processing and injection, one must consider the potential to prepare the recipient site for fat grafting.

To further improve graft take and consistency of temporal fat grafting several strategies could be systematically explored. Graft site preparation is intended to enhance fat graft take (e.g., via external volume expansion, alloplastic material implantation, addition of cell-proliferative factors, microneedling) (36). External volume expansion techniques of the recipient site have been explored which applies negative pressure over the recipient site hoping to stretch and expand it over time (37). One commonly discussed method for achieving this is through external expansion techniques, such as the use of external vacuum-assisted devices (e.g., the Brava system for breasts) before or after fat injection. These devices create a vacuum environment that stretches the recipient tissue, thereby increasing blood flow and tissue oxygenation. This can enhance the integration of the injected fat by facilitating early revascularization—a key determinant for the survival of fat grafts. Tissue expansion is thought to create a more favorable environment for the grafted fat by increasing the available space, improving local perfusion, and reducing mechanical compression that might otherwise impede graft survival. Other adjuncts such as hyperbaric oxygen (HBO) therapy after temporal fat grafting could be explored to enhance graft take. In preclinical studies, the positive effect of HBO on grafted adipose tissue was demonstrated (38). Further systematic studies on the impact of recipient site preparation and other adjuncts to improve graft take in temporal fat grafting could provide valuable insights.

#### Limitations

We acknowledge the limitations due to the reliance on non-instrumental methods for evaluating adipose resorption in many of the studies included in our review. In the future, more randomized controlled prospective studies are necessary to compare different techniques of AFG to restore temporal volume, ideally utilizing more objective quantitative measures like MRI, ultrasound, or other imaging technologies. The follow-up investigations must be more standardized and should be based rather on objective assessment than on various subjective-based scales. Furthermore, the use of standardized patient questionnaires to evaluate patient's satisfaction after temporal augmentation should be established. Furthermore, it is important to note that the reporting of complications in the included articles has not been standardized and remains variable. Therefore, while the risks may be infrequent, they must be acknowledged and managed with precision during the procedure.

## Conclusion

Temporal augmentation with autologous fat grafting is a widely utilized technique for correcting aesthetically displeasing deformities in the temporal area. The available clinical data are notably promising regarding graft survival, complication rates, and patient satisfaction. Although the risk seems low, patients should be fully informed about the potential risks, including adipose embolism, and the importance of selecting experienced practitioners (plastic surgeons) to minimize these risks.

However, this body of research lacks the use of standardized and uniform tools for evaluating clinical outcomes. Thus, further studies, including controlled trials with large sample sizes and consistent evaluation criteria, are essential for enhancing the treatment of temporal hollowness via autologous fat grafts. Continued research is poised to yield long-lasting, satisfactory, and aesthetically appealing outcomes through the application of autologous fat grafting for cosmetic temporal augmentation.
