# Correction: Autologous fat grafting for cosmetic temporal augmentation: a systematic review

**DOI:** 10.3389/fsurg.2026.1870176

**Published:** 2026-06-02

**Authors:** Sahra Nasim, Henna Nasim, Martin Kauke, Ali-Farid Safi

**Affiliations:** 1 Medical Faculty of the University of Bern, Bern, Switzerland; 2 Department of Surgery, Division of Plastic and Reconstructive Surgery, Yale New Haven Hospital, Yale School of Medicine, New Haven, CT, United States; 3 Center for Craniomaxillofacial Surgery, Craniologicum, Bern, Switzerland; 4 Department of Cranio-Maxillofacial Surgery, Inselspital, Bern University Hospital, Bern, Switzerland

**Keywords:** autologous fat grafting, temporal augmentation, facial rejuvenation, volume retention, patient satisfaction, fat transfer techniques, systematic review

Affiliation 4 “Department of Cranio-Maxillofacial Surgery, Inselspital, Bern University Hospital, Berne, Switzerland” was omitted for author Ali-Farid Safi. This affiliation has now been added for author Ali-Farid Safi.

The original version of this article has been updated.

